# 2,4-Dichloro-*N*-cyclo­hexyl­benzamide

**DOI:** 10.1107/S1600536808008131

**Published:** 2008-04-02

**Authors:** Aamer Saeed, Naeem Abbas, Shahid Hussain, Ulrich Flörke

**Affiliations:** a Department of Chemistry, Quaid-i-Azam University, Islamabad, Pakistan; b Department für Chemie, Fakultät für Naturwissenschaften, Universität Paderborn, Warburgerstrasse 100, D-33098 Paderborn, Germany

## Abstract

In the title mol­ecule, C_13_H_15_Cl_2_NO, the cyclohexane ring adopts a chair conformation. The aromatic ring plane is oriented with respect to the N/O/C plane at a dihedral angle of 51.88 (7)°. In the crystal structure, inter­molecular N—H⋯O hydrogen bonds link the mol­ecules into infinite chains along the [010] direction.

## Related literature

For related literature, see: Makino *et al.* (2001 ▶, 2003 ▶); Ho *et al.* (2002 ▶); Zhichkin *et al.* (2007 ▶); Jackson *et al.* (1994 ▶); Capdeville *et al.* (2002 ▶); Manley *et al.* (2002 ▶); Igawa *et al.* (1999 ▶); Jones & Kuś (2004 ▶). For ring conformation puckering parameters, see: Cremer & Pople (1975 ▶).
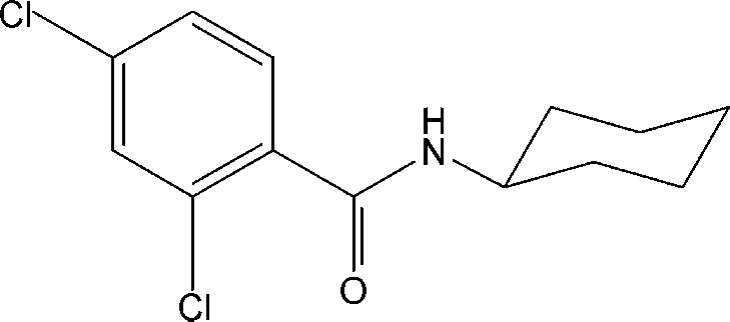


## Experimental

### 

#### Crystal data

C_13_H_15_Cl_2_NO
*M* *_r_* = 272.16
Monoclinic, 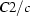

*a* = 26.135 (3) Å
*b* = 4.9144 (6) Å
*c* = 20.449 (2) Å
β = 90.167 (3)°
*V* = 2626.4 (5) Å^3^
*Z* = 8
Mo *K*α radiation
μ = 0.48 mm^−1^
*T* = 120 (2) K
0.48 × 0.17 × 0.12 mm

#### Data collection

Bruker SMART APEX diffractometer
Absorption correction: multi-scan (*SADABS*; Sheldrick, 2004 ▶) *T*_min_ = 0.803, *T*_max_ = 0.945
10950 measured reflections
3141 independent reflections
2389 reflections with *I* > 2σ(*I*)
*R*_int_ = 0.041

#### Refinement

*R*[*F* ^2^ > 2σ(*F* ^2^)] = 0.043
*wR*(*F* ^2^) = 0.110
*S* = 1.02
3141 reflections
154 parameters
H-atom parameters constrained
Δρ_max_ = 0.33 e Å^−3^
Δρ_min_ = −0.21 e Å^−3^

### 

Data collection: *SMART* (Bruker, 2002 ▶); cell refinement: *SAINT* (Bruker, 2002 ▶); data reduction: *SAINT*; program(s) used to solve structure: *SHELXS97* (Sheldrick, 2008 ▶); program(s) used to refine structure: *SHELXL97* (Sheldrick, 2008 ▶); molecular graphics: *SHELXTL* (Sheldrick, 2008 ▶); software used to prepare material for publication: *SHELXL97*.

## Supplementary Material

Crystal structure: contains datablocks I, global. DOI: 10.1107/S1600536808008131/hk2439sup1.cif

Structure factors: contains datablocks I. DOI: 10.1107/S1600536808008131/hk2439Isup2.hkl

Additional supplementary materials:  crystallographic information; 3D view; checkCIF report

## Figures and Tables

**Table 1:** Hydrogen-bond geometry (Å, °)

*D*—H⋯*A*	*D*—H	H⋯*A*	*D*⋯*A*	*D*—H⋯*A*
N1—H1*B*⋯O1^i^	0.88	1.95	2.796 (3)	161

Symmetry code: (i) 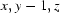.

**Fig. 1.**
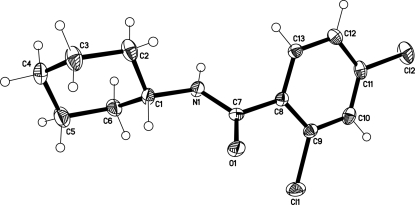
The molecular structure of the title molecule, with the atom-numbering scheme. Displacement ellipsoids are drawn at the 50% probability level.

**Fig. 2.**
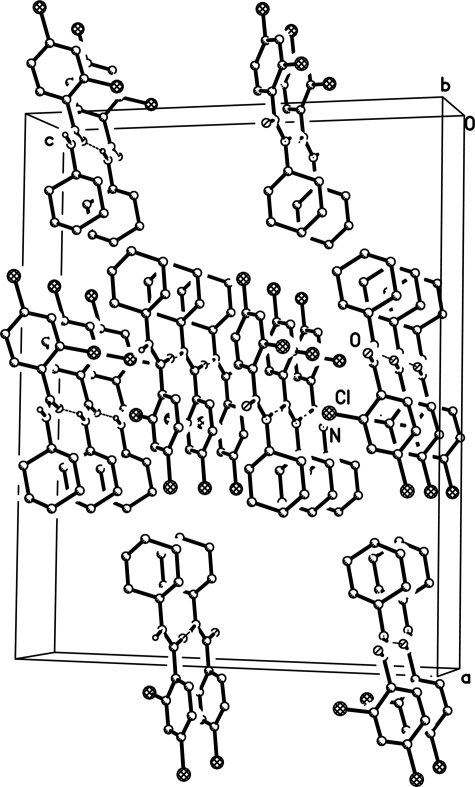
A packing diagram for (I). Hydrogen bonds are shown as dashed lines. H-atoms not involved in hydrogen bonding are omitted for clarity.

## supplementary crystallographic information

### Comment

The benzanilide core is present in compounds with such a wide range of biological activities that it has been called a privileged structure. Benzanilides serve as intermediates towards benzothiadiazin-4-ones (Makino *et al.*,  2003), quinazoline-2,4-diones (Makino *et al.*, 2001), benzodiazepine-2,5-diones (Ho *et al.*,  2002) and 2,3-disubstituted-3H-quinazoline-4-ones (Zhichkin *et al.*, 2007). Benzanilides have established their efficancy as centroid elements of ligands that bind to a wide variety of receptor types. Thus benzanilides containing aminoalkyl groups originally designed as a peptidomimetic have been incorporated in an Arg-Gly-Asp cyclic peptide yielding a high affinity GPIIb/IIIa ligand (Jackson *et al.*,  1994). Imatinib is an ATP-site binding kinase inhibitor and platelet-derived growth factor receptor kinases (Capdeville *et al.*, 2002). Pyridylmethyl containing benzanilides are vascular endothelial growth factor receptor and tyrosine kinase inhibitor (Manley *et al.*,  2002). Furthermore, benzamides have been reported to have activities as acetyl-CoA carboxylase and farnesyl transferase inhibitors (Igawa *et al.*,  1999). We report herein the crystal structure of the title compound, (I).

In the molecule of the title compound, (I), (Fig. 1) the bond lengths and angles are within normal ranges. Ring A (C1–C6) is not planar, having total puckering amplitude, Q_T_, of 0.575 (3) Å. It adopts chair conformation [φ = -177.97 (2)° and θ = 176.74 (3)°] (Cremer & Pople, 1975). Ring B (C8–C13) is, of course, planar and it is oriented with respect to the (N1/O1/C7) plane at a dihedral angle of 51.88 (7)°. The N1—C7—C8—C9 torsion angle is -130.16 (18)°. In N-cyclohexyl-4-(methoxycarbonyl)benzamide (Jones & Kuś, 2004), the corresponding torsion angles are reported as -17.9 (2)° and -45.2 (2)°.

In the crystal structure, intermolecular N—H···O hydrogen bonds (Table 1) link the molecules into infinite chains along the [010] direction (Fig. 2), in which they may be effective in the stabilization of the structure.

### Experimental

A mixture of 2,4-dichlorobenzoyl chloride (65.7 mmol), cyclohexyl amine (86.9 mmol) and pyridine (20 ml) was left at 298 K for 15 h. Then, water (100 ml) was added and the resulting precipitates were collected. Recrystallization of the precipitates from benzene gave the title compound (yield; 75%).

### Refinement

H atoms were positioned geometrically, with N—H = 0.88 Å (for NH) and C—H = 0.95, 0.99 and 1.00 Å for aromatic, methylene and methine H and constrained to ride on their parent atoms, with U_iso_(H) = 1.2U_eq_(C,N).

### Crystal data

**Table:**

C_13_H_15_Cl_2_NO	*F*_000_ = 1136
*M**_r_* = 272.16	*D*_x_ = 1.377 Mg m^−^^3^
Monoclinic, *C*2/*c*	Mo *K*α radiation λ = 0.71073 Å
Hall symbol: -C 2yc	Cell parameters from 978 reflections
*a* = 26.135 (3) Å	θ = 2.5–25.9º
*b* = 4.9144 (6) Å	µ = 0.48 mm^−^^1^
*c* = 20.449 (2) Å	*T* = 120 (2) K
β = 90.167 (3)º	Prism, colorless
*V* = 2626.4 (5) Å^3^	0.48 × 0.17 × 0.12 mm
*Z* = 8	

### Data collection

**Table:**

Bruker SMART APEX diffractometer	3141 independent reflections
Radiation source: sealed tube	2389 reflections with *I* > 2σ(*I*)
Monochromator: graphite	*R*_int_ = 0.041
*T* = 120(2) K	θ_max_ = 27.9º
φ and ω scans	θ_min_ = 1.6º
Absorption correction: multi-scan(SADABS; Sheldrick, 2004)	*h* = −34→34
*T*_min_ = 0.803, *T*_max_ = 0.945	*k* = −6→6
10950 measured reflections	*l* = −26→26

### Refinement

**Table:**

Refinement on *F*^2^	Secondary atom site location: difference Fourier map
Least-squares matrix: full	Hydrogen site location: difference Fourier map
*R*[*F*^2^ > 2σ(*F*^2^)] = 0.043	H-atom parameters constrained
*wR*(*F*^2^) = 0.110	*w* = 1/[σ^2^(*F*_o_^2^) + (0.0561*P*)^2^ + 0.7836*P*] where *P* = (*F*_o_^2^ + 2*F*_c_^2^)/3
*S* = 1.02	(Δ/σ)_max_ < 0.001
3141 reflections	Δρ_max_ = 0.33 e Å^−^^3^
154 parameters	Δρ_min_ = −0.21 e Å^−^^3^
Primary atom site location: structure-invariant direct methods	Extinction correction: none

### Special details

**Table:**

Geometry. All e.s.d.'s (except the e.s.d. in the dihedral angle between two l.s. planes) are estimated using the full covariance matrix. The cell e.s.d.'s are taken into account individually in the estimation of e.s.d.'s in distances, angles and torsion angles; correlations between e.s.d.'s in cell parameters are only used when they are defined by crystal symmetry. An approximate (isotropic) treatment of cell e.s.d.'s is used for estimating e.s.d.'s involving l.s. planes.
Refinement. Refinement of F^2^ against ALL reflections. The weighted R-factor wR and goodness of fit S are based on F^2^, conventional R-factors R are based on F, with F set to zero for negative F^2^. The threshold expression of F^2^ > 2sigma(F^2^) is used only for calculating R-factors(gt) etc. and is not relevant to the choice of reflections for refinement. R-factors based on F^2^ are statistically about twice as large as those based on F, and R- factors based on ALL data will be even larger.

### Fractional atomic coordinates and isotropic or equivalent isotropic displacement parameters (Å^2^)

**Table:**

	*x*	*y*	*z*	*U*_iso_*/*U*_eq_	
Cl1	0.55602 (2)	0.84379 (11)	0.19724 (2)	0.03406 (16)	
Cl2	0.683870 (19)	0.21813 (13)	0.05939 (3)	0.04287 (18)	
O1	0.45551 (5)	0.8154 (2)	0.11531 (7)	0.0276 (3)	
N1	0.43595 (6)	0.3718 (3)	0.12891 (8)	0.0251 (4)	
H1B	0.4481	0.2049	0.1303	0.030*	
C1	0.38119 (6)	0.4124 (4)	0.13939 (10)	0.0246 (4)	
H1A	0.3743	0.6126	0.1403	0.029*	
C2	0.35016 (7)	0.2882 (5)	0.08417 (9)	0.0309 (5)	
H2A	0.3568	0.0901	0.0821	0.037*	
H2B	0.3607	0.3698	0.0421	0.037*	
C3	0.29284 (8)	0.3379 (5)	0.09487 (10)	0.0375 (5)	
H3A	0.2858	0.5356	0.0927	0.045*	
H3B	0.2731	0.2480	0.0595	0.045*	
C4	0.27550 (7)	0.2290 (5)	0.16035 (10)	0.0333 (5)	
H4A	0.2780	0.0280	0.1602	0.040*	
H4B	0.2392	0.2784	0.1673	0.040*	
C5	0.30768 (8)	0.3422 (5)	0.21592 (10)	0.0382 (5)	
H5A	0.2973	0.2548	0.2575	0.046*	
H5B	0.3014	0.5401	0.2200	0.046*	
C6	0.36460 (7)	0.2927 (5)	0.20447 (9)	0.0317 (5)	
H6A	0.3847	0.3769	0.2403	0.038*	
H6B	0.3715	0.0946	0.2048	0.038*	
C7	0.46841 (7)	0.5748 (4)	0.11741 (8)	0.0193 (4)	
C8	0.52252 (6)	0.4894 (3)	0.10407 (8)	0.0179 (4)	
C9	0.56448 (7)	0.6043 (4)	0.13615 (8)	0.0214 (4)	
C10	0.61389 (7)	0.5236 (4)	0.12231 (9)	0.0256 (4)	
H10A	0.6421	0.6031	0.1447	0.031*	
C11	0.62169 (7)	0.3258 (4)	0.07559 (9)	0.0256 (4)	
C12	0.58138 (7)	0.2087 (4)	0.04197 (9)	0.0247 (4)	
H12A	0.5873	0.0740	0.0096	0.030*	
C13	0.53219 (7)	0.2924 (4)	0.05662 (9)	0.0213 (4)	
H13A	0.5042	0.2134	0.0337	0.026*	

### Atomic displacement parameters (Å^2^)

**Table:**

	*U*^11^	*U*^22^	*U*^33^	*U*^12^	*U*^13^	*U*^23^
Cl1	0.0376 (3)	0.0338 (3)	0.0307 (3)	−0.0036 (2)	−0.0058 (2)	−0.0119 (2)
Cl2	0.0182 (2)	0.0630 (4)	0.0474 (3)	0.0106 (2)	0.0028 (2)	0.0024 (3)
O1	0.0274 (7)	0.0119 (7)	0.0436 (8)	0.0027 (5)	0.0016 (6)	0.0005 (6)
N1	0.0181 (8)	0.0111 (7)	0.0463 (10)	0.0031 (6)	0.0046 (7)	−0.0003 (7)
C1	0.0170 (8)	0.0140 (9)	0.0428 (11)	0.0028 (7)	0.0061 (8)	0.0019 (8)
C2	0.0229 (10)	0.0470 (13)	0.0229 (10)	0.0073 (9)	0.0017 (8)	0.0067 (9)
C3	0.0219 (10)	0.0570 (15)	0.0335 (11)	0.0077 (10)	−0.0015 (9)	0.0059 (10)
C4	0.0191 (9)	0.0394 (13)	0.0413 (12)	−0.0002 (9)	0.0046 (8)	0.0033 (10)
C5	0.0295 (11)	0.0534 (15)	0.0318 (11)	0.0005 (10)	0.0091 (9)	−0.0050 (10)
C6	0.0243 (10)	0.0472 (14)	0.0237 (9)	−0.0002 (9)	−0.0003 (8)	−0.0091 (9)
C7	0.0220 (9)	0.0160 (9)	0.0199 (8)	0.0007 (7)	−0.0008 (7)	−0.0017 (7)
C8	0.0202 (8)	0.0142 (8)	0.0195 (8)	0.0000 (7)	0.0002 (7)	0.0041 (7)
C9	0.0254 (9)	0.0178 (9)	0.0209 (8)	−0.0027 (7)	−0.0007 (7)	0.0025 (7)
C10	0.0207 (9)	0.0320 (11)	0.0242 (9)	−0.0058 (8)	−0.0045 (7)	0.0053 (8)
C11	0.0170 (9)	0.0342 (11)	0.0256 (9)	0.0044 (8)	0.0023 (7)	0.0070 (8)
C12	0.0239 (9)	0.0269 (11)	0.0235 (9)	0.0047 (8)	0.0016 (7)	0.0003 (8)
C13	0.0190 (9)	0.0206 (10)	0.0244 (9)	0.0006 (7)	−0.0021 (7)	−0.0008 (7)

### Geometric parameters (Å, °)

**Table:**

Cl1—C9	1.7310 (19)	C4—H4B	0.9900
Cl2—C11	1.7419 (19)	C5—C6	1.526 (3)
O1—C7	1.231 (2)	C5—H5A	0.9900
N1—C7	1.331 (2)	C5—H5B	0.9900
N1—C1	1.461 (2)	C6—H6A	0.9900
N1—H1B	0.8800	C6—H6B	0.9900
C1—C2	1.517 (3)	C7—C8	1.501 (2)
C1—C6	1.519 (3)	C8—C13	1.394 (2)
C1—H1A	1.0000	C8—C9	1.396 (2)
C2—C3	1.534 (3)	C9—C10	1.381 (3)
C2—H2A	0.9900	C10—C11	1.378 (3)
C2—H2B	0.9900	C10—H10A	0.9500
C3—C4	1.513 (3)	C11—C12	1.382 (3)
C3—H3A	0.9900	C12—C13	1.383 (3)
C3—H3B	0.9900	C12—H12A	0.9500
C4—C5	1.518 (3)	C13—H13A	0.9500
C4—H4A	0.9900		
			
C7—N1—C1	123.28 (15)	C4—C5—H5B	109.3
C7—N1—H1B	118.4	C6—C5—H5B	109.3
C1—N1—H1B	118.4	H5A—C5—H5B	108.0
N1—C1—C2	110.95 (16)	C1—C6—C5	110.70 (17)
N1—C1—C6	110.96 (16)	C1—C6—H6A	109.5
C2—C1—C6	110.05 (15)	C5—C6—H6A	109.5
N1—C1—H1A	108.3	C1—C6—H6B	109.5
C2—C1—H1A	108.3	C5—C6—H6B	109.5
C6—C1—H1A	108.3	H6A—C6—H6B	108.1
C1—C2—C3	110.49 (17)	O1—C7—N1	123.48 (16)
C1—C2—H2A	109.6	O1—C7—C8	121.36 (16)
C3—C2—H2A	109.6	N1—C7—C8	115.11 (15)
C1—C2—H2B	109.6	C13—C8—C9	117.66 (16)
C3—C2—H2B	109.6	C13—C8—C7	119.55 (15)
H2A—C2—H2B	108.1	C9—C8—C7	122.76 (15)
C4—C3—C2	111.41 (16)	C10—C9—C8	121.43 (17)
C4—C3—H3A	109.3	C10—C9—Cl1	117.70 (14)
C2—C3—H3A	109.3	C8—C9—Cl1	120.82 (14)
C4—C3—H3B	109.3	C11—C10—C9	119.00 (17)
C2—C3—H3B	109.3	C11—C10—H10A	120.5
H3A—C3—H3B	108.0	C9—C10—H10A	120.5
C3—C4—C5	111.50 (18)	C10—C11—C12	121.64 (17)
C3—C4—H4A	109.3	C10—C11—Cl2	119.08 (14)
C5—C4—H4A	109.3	C12—C11—Cl2	119.28 (15)
C3—C4—H4B	109.3	C11—C12—C13	118.43 (18)
C5—C4—H4B	109.3	C11—C12—H12A	120.8
H4A—C4—H4B	108.0	C13—C12—H12A	120.8
C4—C5—C6	111.40 (17)	C12—C13—C8	121.83 (17)
C4—C5—H5A	109.3	C12—C13—H13A	119.1
C6—C5—H5A	109.3	C8—C13—H13A	119.1
			
C7—N1—C1—C2	113.7 (2)	N1—C7—C8—C9	−130.16 (18)
C7—N1—C1—C6	−123.64 (19)	C13—C8—C9—C10	−1.0 (3)
N1—C1—C2—C3	−178.66 (16)	C7—C8—C9—C10	−179.29 (16)
C6—C1—C2—C3	58.1 (2)	C13—C8—C9—Cl1	−178.28 (13)
C1—C2—C3—C4	−56.3 (2)	C7—C8—C9—Cl1	3.4 (2)
C2—C3—C4—C5	54.1 (3)	C8—C9—C10—C11	0.2 (3)
C3—C4—C5—C6	−54.1 (3)	Cl1—C9—C10—C11	177.62 (14)
N1—C1—C6—C5	178.53 (16)	C9—C10—C11—C12	0.6 (3)
C2—C1—C6—C5	−58.3 (2)	C9—C10—C11—Cl2	−178.55 (14)
C4—C5—C6—C1	56.2 (2)	C10—C11—C12—C13	−0.7 (3)
C1—N1—C7—O1	0.9 (3)	Cl2—C11—C12—C13	178.49 (14)
C1—N1—C7—C8	−176.58 (16)	C11—C12—C13—C8	−0.1 (3)
O1—C7—C8—C13	−126.00 (18)	C9—C8—C13—C12	0.9 (3)
N1—C7—C8—C13	51.5 (2)	C7—C8—C13—C12	179.29 (17)
O1—C7—C8—C9	52.3 (2)		

### Hydrogen-bond geometry (Å, °)

**Table:**

*D*—H···*A*	*D*—H	H···*A*	*D*···*A*	*D*—H···*A*
N1—H1B···O1^i^	0.88	1.95	2.796 (3)	161

Symmetry codes: (i) *x*, *y*−1, *z*.
